# Facial Papulopustular Eruption during the COVID-19 Pandemic in Patients Treated with EGFR Inhibitors

**DOI:** 10.1155/2024/8859032

**Published:** 2024-01-11

**Authors:** Eleonora De Luca, Pietro Sollena, Lucia Di Nardo, Ettore D'Argento, Emanuele Vita, Giampaolo Tortora, Ketty Peris

**Affiliations:** ^1^Dermatologia, Dipartimento Universitario di Medicina e Chirurgia Traslazionale, Università Cattolica del Sacro Cuore, Rome, Italy; ^2^Dermatologia, Dipartimento di Scienze Mediche e Chirurgiche, Fondazione Policlinico Universitario A. Gemelli IRCCS, Rome, Italy; ^3^Oncologia, Dipartimento di Scienze Mediche e Chirurgiche, Fondazione Policlinico Universitario A. Gemelli IRCCS, Rome, Italy

## Abstract

Papulopustular rash (PPR) is the most frequent cutaneous adverse event during treatment with epidermal growth factor receptor inhibitors (EGFRis). Although often mild in severity, it can impair patients' quality of life and may also be a reason for discontinuing or changing the dose of the antineoplastic treatment. During COVID-19 pandemics, the use of surgical masks drastically increased and it had an impact on the face skin microenvironment, favoring the worsening of dermatological pathologies. We reported the relapse of PPR in patients treated with EGFR inhibitors who consistently wore face masks (>6 hours/day). All the patients developed the PPR within 6 months of starting mask use. Compared to the PPR occurred previously, after mask use, the skin eruption was more severe and affected mainly those regions of the face which came into contact with the mask. Patients received topical or systemic treatment, obtaining complete response in 65.7% of the cases. The establishment of an early treatment for the PPR allows continuing the oncologic treatment, without any suspension which could result in a decreased oncologic outcome. In conclusion, when using these devices, it is recommended to use special precautions, particularly in oncologic patients, by using a daily prophylactic skincare and replacing masks regularly with regular and frequent breaks.

## 1. Introduction

The epidermal growth factor receptor (EGFR) is a tyrosine kinase receptor involved in cell proliferation, survival, and differentiation in both normal and cancer cells [1]. EGFR inhibitors (EGFRis) are targeted agents which provided a major advance in the management of many malignant tumors including colorectal cancer [2], head and neck squamous cell carcinoma [3], nonsmall-cell lung cancer [4], breast cancer [5], and pancreatic cancer [6].

EGFR inhibitors demonstrate a high efficacy in treating aggressive tumors; however, they are also associated with frequent cutaneous side effects (50–90% of the patients) [7, 8] as well as adverse events occurring in the gastrointestinal tract (mainly diarrhea and stomatitis/mucositis), hepatotoxicity, interstitial lung diseases, ocular toxicity, and electrolyte imbalances (mostly hypomagnesaemia) [9, 10].

Skin manifestations include papulopustular lesions (>70%), dry skin, pruritus, paronychia, pyogenic granulomas, hair and nail abnormalities (mostly trichomegaly) and rarely severe bullous dermatitis, Stevens–Johnson syndrome, and toxic epidermal necrolysis [5, 11–13]. The severity of cutaneous reactions is assessed according to the National Cancer Institute: Common Terminology Criteria for Adverse Events version 5.0 (NCI CTCAE v5.0) scale [14]. Skin reactions are often mild but can be painful and impair patients' emotional well-being and quality of life [15]. In case of severe cutaneous reactions (CTCAE grade 3 or higher), it may also be necessary to discontinue the oncologic therapy or decrease its dosage [16–18]. It has also been hypothesized that skin adverse events may be considered a marker of the efficacy of the therapy and evidence of the antitumor activity. Some studies report higher response rates and longer survival times among patients who developed skin reactions compared to patients who did not develop any skin adverse event [19–21]. Cutaneous reactions are mainly managed with topical treatments, including moisturizers, antibiotics, and corticosteroids [22]. Prophylactic management with hydrophilic cream, antibiotics, and sunscreen reduces the incidence of dermatological toxicity associated with EGFR-targeted agents by 42–77%, without affecting the antitumor efficacy and improving patients' quality of life [13, 23].

The papulopustular reaction (PPR) usually starts within 3 weeks after the initiation of the EGFR inhibitors treatment [20, 24]. Patients first develop erythema and edema which are followed by papules, pustules, and nodules in the absence of comedones [25, 26]. The PPR mainly affects the sebaceous areas on the body; it is mostly located on the face (>80%), trunk (>50%), scalp (<20%), and extremities (>20%) [27]. The pathogenetic mechanism underlying the papulopustular manifestation has been related to the inhibition of the EGFR of the epidermal keratinocytes resulting in apoptosis and release of inflammatory cytokines [28, 29].

In 2020, during COVID-19 pandemics, the use of surgical and FFP2 masks drastically increased, changing the face-skin equilibrium and inducing an increase of temperature and humidity, dehydration, transepidermal water loss, increased pH, erythema, and sebum dysregulation [30–32]. Higher temperature and humidity are associated with an increase of sebum excretion, which has a role in acne pathogenesis and, therefore, can cause acne flares [33]. This cutaneous microclimate led to a skin dysbiosis (increasing number of *Cutibacterium acnes and Desmodex folliculorum*) and, therefore, to an inflammatory response [34–36]. Due to these changes, the use of masks was recently associated to the onset of mask-related acne, a subtype of acne mechanica related to the friction of the textile on the skin [37], and the worsening of other dermatological pathologies such as rosacea, eczema, and perioral dermatitis [38, 39].

The effect of mask use in patients treated with EGFR inhibitors has not been explored yet. In this study, we reported and characterized the relapse of PPR in patients treated with EGFR inhibitors who consistently wore face masks during COVID-19 pandemics.

## 2. Materials and Methods

We performed a retrospective study to evaluate the presence or the relapse of the papulopustular reaction located on the face in patients treated with EGFR inhibitors who wore face masks for a consistent amount of time. Patients in treatment with EGFRi afferent to the Department of Oncology of Agostino Gemelli Foundation (FGP) were consecutively enrolled from March 2020 to March 2021.

Inclusion criteria were patients >18 years old, treatment with EGFR inhibitor, and the use of face masks for at least 6 hours a day minimum 1 month (range 1–6). Exclusion criteria consisted of the presence of papulopustular manifestation before starting EGFRi, patients affected by pilosebaceous unit congenital diseases, and chronic assumption of topical and/or systemic antibiotics.

Data related to the characteristics of the patients, type of cancer and oncologic treatment, PPR, use of mask, and previous adverse dermatological reactions were collected at the time of dermatological observations (Table 1).

We assessed the PPR severity with the CTCAE v5.0 scale [14] and the Multinational Association for Supportive Care in Cancer (MASCC) EGFR Inhibitor Skin Toxicity Tool (MESTT) scale [40]. The CTCAE scale grades from 1 to 5, from the mildest to the most severe, based on the percentage of affected skin and on the presence of cutaneous symptoms. The MESTT is an EGFR inhibitor dermatological adverse event-specific grading scale proposed by the MASCC toxicity group to better describe the PPR; it includes objective parameters as the number of papules and pustules and erythema and edema, as well as symptoms and quality of life measures.

Semiquantitative data were analyzed by Student's *t*-test or by medians with the Mann–Whitney test. Univariate analysis by *χ*^2^ test or by Fisher's exact test was used to test the significance of cutaneous adverse events frequency and PPR severity according to clinic-pathological characteristics of patients and to the specific type of EGFRi administered.

We used a simplified MESTT grading combining MESTT 1a/1a into the MESTT grading 1 category, 2a/2b into the MESTT grading 2 category, and 3a/3b into the MEST grading 3 category.

To assess the difference of localization and severity of the PPR before and after mask use, we used a Wilcoxon signed rank test for matched-pairs' ordinal data. The association among clinical outcome (complete response vs. partial response), clinic-pathological characteristics, and PPR treatment was evaluated with univariable and multivariable logistic regressions and presented through odds ratios (ORs) with 95% confidence intervals (CIs). Both a stepwise selection and the bivariate Wald's model were used. The alpha level for all analyses was set to *p* < 0.05. All statistical analyses were performed using STATA/BE v.17.0.

## 3. Results

Thirty-five patients comprising 19 females (54%) and 16 males (46%) treated with EGFRi were included in the study. Internal malignancies included nonsmall-cell lung cancer in 16/35 (46%) patients, breast cancer (1/35, 3%), gastrointestinal cancer (14/35, 40%), and head and neck squamous cell carcinoma (4/34, 11%). 9 patients (26%) were treated with cetuximab, 1 patient (3%) with erlotinib, 7 patients (20%) with afatinib, 2 patients (6%) with osimertinib, 7 patients (20%) with gefinitinib, 8 patients (23%) with panitumumab, and 1 patient (3%) with vandetanib. Other ongoing oncologic treatments included BRAF inhibitor in 1 patient (3%) and FOLFIRI chemotherapy protocol (folinic acid, fluorouracil, and irinotecan) in 6 patients (17%).

**Figure 1:**
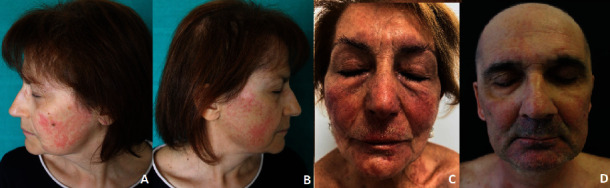
Relapse of papulopustular rash after mask use, localized on cheeks and chin.

The papulopustular manifestation was localized on the entire face in 46% of the patients (16/35), exclusively on the cheeks and chin in 37% of the patients (13/35), and on both face and body in 17% of the patients (6/35) (Figure 1). Severity according to the CTCAE scale was grade 1 in 20% of the patients (17/35), grade 2 in 60% (21/35), and grade 3 in 20% (7/35). Evaluated with the MESTT scale, 14% (5/35) of the cases were grade 1b, 3% (1/35) grade 2a, 51% (18/35) grade 2b, 11% (4/35) grade 3a, and 20% (7/35) grade 3b. Symptoms included pruritus (80% of the patients, 28/35), dry skin (34%, 12/35), and burning (86%, 30/35).

Based on the start date of mask use reported by the patients, all patients developed the PPR within 6 months of starting mask use; 66% (23/35) of them developed the papulopustular reaction in the first 3 months.

Patients were given topical treatment in all cases although 7/35 (20%) patients needed to add systemic antibiotics (doxycycline 100 mg daily). Topical therapies included potency steroids in 8/35 patients (23%), mild potency steroids in 2/35 (6%), topical steroids associated with antibiotics in 20/35 (57%), emollients in 2/35 (6%), and topical retinoids associated with steroids in 3/35 (9%).

All the patients improved with the treatment; 34% (12/35) obtained a partial response and 66% (23/35) a complete response (remission of the PPR). The severity of PPR required a modification of the oncologic treatment in 6 patients (6/35, 17%); no patient needed the discontinuation of oncologic treatment. The oncologic treatment outcome at the time of dermatological examination was partial response in 69% of the patients (25/35) and complete response in 17% (6/35).

**Figure 2:**
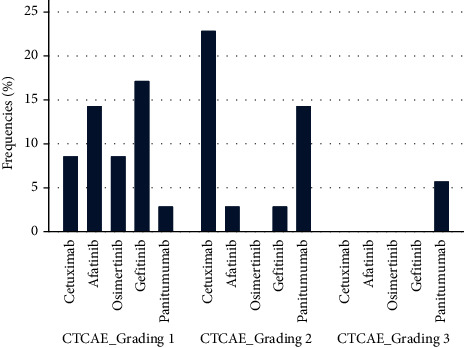
PPR grading prior to facemask use and the type of EGFRi administered.

Medical history revealed a previous skin papulopustular eruption caused by EGFRi, mainly located on both face and body (31/35, 89%). Previous PPR severity assessed with the CTACAE scale was grade I in 18 patients (18/35, 51%), grade 2 in 15 patients (15/35, 43%), and grade 3 in 2 patients (2/35, 6%). Evaluated with the MESTT scale, 23% (8/35) of the previous PPR was grade 1a, 29% (10/35) was grade 1b, 26% (9/35) was grade 2a, 17% (6/35) was grade 2b, and 6% (2/35) was grade 3a. By evaluating the relationship between PPR grading prior to face mask use and the type of EGFRi administered (cetuximab, afatinib, osimertinib, gefitinib, and panitumumab), we found a significant higher frequency of CTCAE grade 2 PPR (MESTT grade 2a/2b) among patients undergoing panitumumab and cetuximab than other EGFRi, while grade 3 PPR occurred only in patients receiving panitumumab (*p*=0.007) (Figure 2). Instead, combination therapy with BRAFi and/or FOLFIRI did not influence PPR severity (*p*=0.062). Other skin adverse events related to EGFRi treatment were observed in 8 patients (all grade 1) as follows: 5 paronychia, 1 pyogenic granuloma, 1 trichomegaly, and 1 ungual dystrophy. No patient referred personal history of previous dermatoses.

We analyzed the difference of the PPR developed before and during the use of the mask during the pandemics. A Wilcoxon signed rank test indicates that the PPR severity after face mask use was greater than prior to using it (*z* = −4.0, *p*=0.0001 and *z* = −4.4, *p* < 0.0001, for CTCAE and MESTT scale, respectively). We also investigated the PPR localization pre- and postface mask use, showing a significant difference (*z* = −5.18, *p* < 0.0001). The most frequent site of PP manifestation during the mask use was the entire face (16/35, 82.9%), followed by cheeks and chin (13/35, 37.14%), while prior to face mask use, PPR was mostly localized on both face and body (31/35, 88.57%) and on other body sites (4/35, 11.43%).

Topical (steroid cream, combo steroid + antiseptic/antibiotic cream, emollient cream, and topical retinoids + steroids) and systemic treatments (antibiotics) have been used for the management of PPR, obtaining complete response in 65.7% (23/35) of the patients. We performed univariable and multivariable logistic regression analysis to evaluate the association among clinical outcome (CR versus PR), clinic-pathological characteristics, and treatment (type of topical/systemic treatment, type of combo therapy, type of EGFRi, PPR localization, PPR severity, gender, and age). PPR severity, both assessed through MESTT and CTCAE scales, was found to be the only independent predictor of partial response, irrespective of gender, age, and EGFRi administered (Table 2). Logistic regression was used for univariable and multivariable analyses; both a stepwise selection and the bivariate Wald's model were used.

## 4. Discussion

The results of our study indicate that wearing face mask had a significant effect in relapsing and worsening the severity of PPR as an adverse event during EGFRi treatment.

During the COVID-19 pandemic, the use of face masks increased among healthcare professionals and general population, helping to slow the spread of the virus. Long-time mask wearing was associated to several facial dermatosis, including irritant and contact dermatitis, seborrheic dermatitis, rosacea, and acne. The change of temperature, humidity, and the cutaneous microclimate is responsible of triggering flares of acne and rosacea, as reported in several studies [41–44]. Topical treatment with salicylic acid, benzoyl peroxide, topical retinoids, or ivermectine was recommended to treat mild cases. Moderate-severe cases can be treated with systemic antibiotics as tetracycline [45, 46].

The oncologic patients included in our study, treated with EGFR inhibitors, developed a relapse of the papulopustular reaction as cutaneous adverse event. The prolonged use of face mask (>6 hours/day) determined the relapse of a PPR with higher severity and with the specific localization on the face. Compared to the PPR occurred previously, located in 89% of the cases on both face and body, during the COVID-19 pandemic, the skin eruption affected mainly those regions of the face which came into contact with the mask (lower third of the face). The PPR manifested all over the face in 46% of the enrolled patients while in 37% of the patients, it was located only on the cheeks and chin area. The alteration of the microenvironment, temperature, and humidity induced a skin dysbiosis which resulted in a higher severity of the skin adverse event, assessed with both the CTCAE scale and the MEST scale. The friction of the textile on the skin and the subsequent increase of the inflammatory response were probably the cause of the worsening symptomatology associated to the PPR. Patients complained pruritus (80% of patients), xerosis (34.3%), and burning sensation (85.7%), especially while wearing face masks. All the patients developed the PPR within 6 months of the start of mask use and 65.7% within 3 months. Topical treatment with emollients, steroids, and antibiotics or systemic treatment with antibiotics resulted in a clinical benefit in all our patients and in a complete remission of the eruption in 65.7% of the cases. The establishment of an early treatment for the PPR allows continuing the oncologic treatment without any suspension that could result in a decreased oncologic outcome.

The use of face masks is considered one of the main strategies for COVID-19 prevention. In case of use of these protective devices, it is recommended to use special precautions. We recommend daily prophylactic skincare with gentle cleanser and noncomedogenic emollients. Masks should be replaced regularly and hands should be washed before and after touching the mask. Regular breaks from the mask may reduce pressure and irritation and, therefore, the relapse of PPR or other facial dermatoses.

## 5. Conclusions

In conclusion, this study highlights the significant impact of prolonged face mask use during the COVID-19 pandemic on patients treated with EGFRi, leading to the relapse and exacerbation of PPR as a cutaneous adverse event. The altered microenvironment caused by mask use, including changes in temperature and humidity, induced skin dysbiosis and heightened severity of the PPR, particularly localized on the face in regions in contact with the mask. The study emphasizes the importance of early and effective management, including topical and systemic treatments, to alleviate PPR symptoms and allow patients to continue oncologic treatment without interruptions. The findings underscore the need for prophylactic skincare measures for individuals undergoing EGFRi therapy and regular breaks from mask use to mitigate the risk of dermatological complications.

## Figures and Tables

**Table 1:** Characteristics of the patients, type of cancer and oncologic treatment, PPR, use of mask, and previous adverse dermatological reactions.

		Number of patients (percentage)
Sex	Female	19 (54.3%)
	Male	16 (45.7%)

Primary cancer	NSCLC	16 (45.7%)
	Breast cancer	1 (2.9%)
	Gastrointestinal cancer	14 (40%)
	HN SCC	4 (11.4%)

Anti-EGFR drug	Cetuximab	9 (25.7%)
	Erlotinib	1 (2.9%)
	Afatinib	7 (20.0%)
	Osimertinib	2 (5.7%)
	Gefitinib	7 (20.0%)
	Panitumumab	8 (22.9%)
	Others	1 (2.9%)

Combined therapy	BRAF inhibitors	1 (2.9%)
	FOLFIRI	6 (17.1%)

PPR localization	Cheeks and chin	13 (37.1%)
	All face	16 (45.7%)
	Face and body	6 (17.1%)

Symptoms associated to the PPR	Puritus	28 (80%)
	Xerosis	12 (34.3%)
	Burning	30 (85.7%)

PPR MEST grading	Grade 1b	5 (14.3%)
	Grade 2a	1 (2.9%)
	Grade 2b	18 (51.4%)
	Grade 3a	4 (11.4%)
	Grade 3b	7 (20.0%)

PPR CTCAE grading	Grade 1	17 (20%)
	Grade 2	21 (60%)
	Grade 3	7 (20%)

Time to PPR since mask use	1 month	4 (11.4%)
	2 months	7 (20%)
	3 months	12 (34.3%)
	4 months	6 (17.1%)
	5 months	4 (11.4%)
	6 months	2 (5.7%)

Topical treatment	High potency steroid cream	8 (22.9%)
	Mild potency steroid cream	2 (5.7%)
	Combo steroid + antiseptic/antibiotic cream	20 (57.1%)
	Emollient cream	2 (5.7%)
	Topical retinoids + steroids	3 (8.6%)

Systemic treatment	Antibiotics (doxycycline)	7 (20%)

Cutaneous outcome	Partial response	12 (34.3%)
	Complete response	23 (65.7%)

Oncologic treatment	Modification	6 (17.1%)
	Discontinuation	0 (0%)

Oncological treatment outcome	Partial response	24 (65.6%)
	Complete response	6 (17.1%)
	No response	5 (14.3%)

Previous PPR localization	Face and body	31 (88.6%)
	Others	4 (11.4%)

Previous PPR MEST grading	Grade 1a	8 (22.9%)
	Grade 1b	10 (28.6%)
	Grade 2a	9 (25.7%)
	Grade 2b	6 (17.1%)
	Grade 3a	2 (5.7%)

Previous PPR CTACAE grading	Grade 1	18 (51.4%)
	Grade 2	15 (42.9%)
	Grade 3	2 (5.7%)

Other skin adverse event	Paronychia	5 (4.3%)
	Pyogenic granuloma	1 (2.9%)
	Trichomegaly	1 (2.9%)
	Ungueal dystrophy	1 (2.9%)

Grade of other skin adverse events	Grade 1	8 (100%)

Personal history of dermatoses	None 35	35 (100%)

NSCLC: nonsmall-cell lung cancer. HN SCC: head and neck squamous cell carcinoma. EGFR: epidermal growth factor receptor. PRP: papulopustular reaction. FOLFIRI: folinic acid-fluorouracil-irinotecan regimen. MEST: Multinational Association for Supportive Care in Cancer (MASCC) EGFR inhibitor skin toxicity tool. CTCAE: National Cancer Institute: Common Terminology Criteria for Adverse Events.

**Table 2:** Multivariable logistic regression model describing the association of clinicopathological and treatment characteristics with clinical response (prob > chi2 = 0.0048; pseudo *R*^2^ = 0.3740).

Partial response	OR (odds ratio)	Std. err.	*P* > |*z*|	95% CI
CTCAE grading 1	Ref			
CTCAE grading 2	0.011	0.017	**0.006**	0.0004–0.2664
CTCAE grading 3	0.003	0.006	**0.006**	0.00004–0.174
Female	Ref			
Male	0.09	0.126	0.091	0.005–1.4707
Age	0.94	0.049	0.249	0.8502–1.0429
EGFRi				
Cetuximab	Ref			
Afatinib	1.73	2.305	0.682	0.1263–23.623
Osimertinib	1.38	3.902	0.910	0.0053–355.86
Gefitinib	2.21	4.445	0.694	0.0428–114.04
Panitumumab	4.25	5.667	0.278	0.4964–820.65

MEST grading 1 (1a/1b)^*∗*^	Ref			
MEST grading 2 (2a/2b)	0.10	0.136	0.092	0.0067–1.4631
MEST grading 3 (3a/3b)	0.01	0.017	**0.009**	0.00031–0.3114
Female	Ref			
Male	0.29	0.339	0.289	0.0314–2.7972
Age	0.96	0.047	0.362	0.8683–1.0528
EGFRi				
Cetuximab	Ref			
Afatinib	1.68	2.262	0.701	0.1196–23.5546
Osimertinib	0.57	1.123	0.776	0.0121–26.9324
Gefitinib	1.61	2.687	0.775	0.0611–42.4065
Panitumumab	1.40	6.656	0.151	0.15100–217892.7

Bold values are statistically significant.

## Data Availability

The data used to support the findings of this study are available from the corresponding author upon request.
